# Seventeenth Annual Meeting of Northern Ohio Dental Association

**Published:** 1876-10

**Authors:** L. C. Kilsey


### Proceedings of Societies.

### SEVENTEENTH ANNUAL MEETING OF NORTHERN OHIO DENTAL ASSOCIATION.

*Held at Cleveland, Ohio, commencing Tuesday, June 13, 1876.*

### MORNING SESSION.

President E. J. Waye being absent, the meeting was called to order at eleven o'clock a. m. by Vice-President J. F. Siddall.

The minutes of the last meeting were read and approved.

Reports of officers then being in order, the treasurer, J. E. Robinson, reported that there was a balance in his hands of four dollars and thirty-six cents.

The corresponding secretary and chairman of the executive committee, Chas. Buffett, reported an order of business, and the following subjects for discussion:

First, Treatment of Exposed Dental Pulps.

Second, Replanting and Transplanting Natural Teeth.

Third, Methods and Appliances for correcting Dental Irregularities.

Fourth, The different Bases for Artificial Dentures.

Fifth, Miscellaneous.

The board of examiners not being present, the chair appointed S. P. Hildrith, L. Buffett and F. S. Whitslar to act as examiners until the election of a new board.

Dr. J. E. Robinson proposed the name of J. M. Whitney as a suitable person to become a member of the association.

A motion was then made that the rule of the association requiring candidates to go before the board of examiners to be examined as to qualifications, be suspended in the case of Dr. J. M. Whitney. Carried.

On motion Dr. Whitney was then admitted to membership in the association.

The name of Dr, W. C. Stewart, of Elyria, was then proposed as a suitable person to become a member of the association.

On motion, the rule of the association requiring candidates to be examined before being admitted to membership, was suspended in the case of Dr, Stewart.

A motion was then made that Dr. W. C. Stewart be received into membership in the association. Carried.

Dr. Chas. Buffett read a communication from Pres. E. J. Waye, of Sandusky, which on motion was placed on file.

On motion, the association adjourned to meet at two o'clock p. m.

AFTERNOON SESSION.

Meeting called to order at two o'clock p. m., by Vice-President J. F. Siddall.

Dr. Chas. Buffett, corresponding secretary, presented a bill for stationery, etc., which, on motion, was accepted, and an order drawn on the treasurer for the amount.

Dr. S. P. Hildreth, chairman of the board of examiners, reported that Dr. David Gibbons had passed a satisfactory examination before the board, and was duly recommended as a suitable person to become a member of the association.

A motion was made that Dr. David Gibbons be received into membership of the association. Carried.

Dr. J. M. Whitney, who had for several years practiced dentistry in the Sandwich Islands, read a very interesting essay before the association relative to the teeth of the inhabitants of the islands. The Doctor illustrated his subject by a variety of skeletons, which he had obtained while on the islands, and showed many peculiarities in dental development and in the diseases of the teeth, which were seldom met with in this country.

On motion, the thanks of the association were tendered to Dr. Whitney for his essay.

Dr. S. P. Hildreth, chairman of the board of examiners, recommended Drs. E. E. Rodgers, of Hudson, and J. W. Lyder, of Akron, as suitable persons to become members of the association.

On motion, Drs. E. E. Rodgers and J. W. Lyder were received into membership.

The subject, Treatment of Exposed Dental Pulps, was then taken up.

Dr. F. S. Whitslar opened the discussion by saying that a great many means had been resorted to for the purpose of preserving dental pulps. In his own practice he had sometimes been successful; some times he had failed in his efforts to preserve the pulps of teeth. In some constitutions, especially in those of a scrofulous diathesis, or in persons where there is a tendency to the formation of pus, it was almost impossible to preserve an exposed pulp. He had used all kinds of cappings, but more recently had used Husband's court plaster exclusively. It was a non-irritant and easily applied. Would recommend this capping to the profession.

Dr. L. Buffett: As a general rule, pulps may and should be saved when there is recent exposure, or in cases where there is but little inflammation,

It is a delicate matter to apply any kind of a capping to an exposed pulp so as to cause no irritation.

In applying a capping it must be put in direct contact with the pulp. The air must be excluded; there must be no space for the formation and retention of gases.

Dr. David Gibbons remarked, that this had been a subject of much thought and interest to him; he had had some personal experience in the treatment of an exposed and inflamed dental pulp in one of his own teeth. The tooth had at times caused him much pain, but by careful management he had succeeded in allaying the inflammation; had the pulp capped, the tooth filled, and it had proved useful to him ever since.

Further and important remarks of Dr. Gibbons the secretary was unable to record, on account of his attention being called to other matters.

Dr. Whitney stated, that in his practice he had often had good results in the treatment of exposed pulps, by the use of nitric acid; he would touch the pulp with the acid, then thoroughly wash out the cavity with soda water, and fill.

Dr. C. R. Butler: It is a very important matter to preserve the pulps of teeth; they were placed in the teeth for some good and wise purpose, and should be kept there if possible. He would preserve them if only for a short time. Great care should be taken in the treatment of exposed dental pulps.

Several conditions follow an exposed dental pulp; would mention one, to-wit: the calcified condition of the pulp, causing irritation and inflammation. The best protection to an exposed pulp is to cover it with a non-irritating substance, such as Hill's Stopping. In applying any capping, care should be taken that it does not press or infringe too much upon the pulp.

In all cases where the pulp is destroyed, sooner or later the integrity of the crown is destroyed. My practice is to keep the pulp alive as long as possible. Have not used any escharotics in destroying dental pulps but in one instance for three years. I make a large and free opening, and remove the pulp mechanically.

Further discussion of this topic was passed, and the next subject, Replanting and Transplanting Natural Teeth, was taken up.

This being the first time this important subject was ever presented to the association, it elicited much enthusiasm and discussion. It opened a new and interesting field, and if the replanting of the natural teeth can, as a general thing, be made a successful operation, a new and important progressive step has been taken in dentistry.

Dr. C. B. Knowlton, being called upon to open the discussion, stated that during his practice he had given much thought and attention to replanting the natural teeth. He had replanted some forty or fifty teeth, and in almost every instance with marked success to himself and patients. He would mention only two or three cases, as these would fully illustrate his manner of proceeding, and afford a key to other cases.

One case treated was where the four superior incisors were knocked out of place. These were taken out of the mouth, the apex of each root amputated, the nerve removed, and the nerve canal filled from the apex. The sockets were thoroughly cleansed, the teeth replaced and held firmly in position by warming a band of gutta percha, molding it to the replaced teeth, and then ligating it to the other teeth. This band would serve as a splint or support to the teeth, until the healing process was nearly or wholly completed. In this case very little, if any periostitis followed The teeth soon became firm and useful.

In another case the superior central incisors were knocked out, and the alveolar process badly broken.

This case was treated very much in the same manner, as the one already described, and what is rather remarkable in this case there was no sloughing, and in a short time there seemed to be a firm reunion of the fractured process.

Had effectually cured several cases of alveolar abscess, by extracting the effected teeth, cutting off a small portion of the end of the root, cleansing the cavity thoroughly with alcohol, and carefully replacing the teeth. The root was amputated for the purpose of avoiding the pressure of sense of elongation in the process of healing.

Alcohol he regarded as a specific for alveolar abscess.

Dr. C. R, Butler: What Dr. Knowlton claims in treating and curing alveolar abscess, can be accomplished in another way, simply by cutting through the process at the apex of the root. The abscess can be thoroughly treated in this way and effectually cured.

Dr. Hildreth, inquired of Dr. Knowlton how long after the extraction of a tooth, could it remain out of the mouth, and then be replaced, to ensure success in the operation?

Dr. Knowlton: For several hours by keeping it in warm water.

Dr. J. W. Lyder then being called upon, gave a very interesting account of the replanting of one of his teeth.

He said that for several years his left superior central incisor had been loose and troublesome, he finally concluded to try the experiment of replanting it. Upon the removal of the tooth he found that about two-thirds of the root was covered with little particles of salivary calculus; these were taken off, the root thoroughly washed with a solution of carbolic acid and chloroform, the tooth was then replaced, and in a short time became firm and serviceable.

Dr. Whitney remarked that he had never tried the experiment of replanting teeth, but knew of one case of tetanus and death as the result of such an operation and this had deterred him from any attempt in that line of practice.

Dr. Whitslar said that in the matter of replanting teeth his experience extended to only one case, the result of which was very satisfactory. What he regarded as quite remarkable in this single case, which he had treated, was the apparent reunion of the nerve, as afterwards the tooth was quite sensitive in preparing it for a filling.

Dr. Slosson gave quite an interesting account of his experience in replanting three teeth, for three persons. In two of these cases the experiment was a success, in the other a failure.

On motion the association adjourned to meet at 7½ o'clock p. m.

EVENING SESSION.

Meeting called to order at half past seven o'clock p. m., Vice President J. F. Siddall in the chair.

The next subject for discussion, Methods and Appliances for Correcting Dental Irregularities, was taken up.

Dr. J. E. Robinson, remarked that he had been very much annoyed in the matter of correcting dental irregularities. He had used almost every appliance. Lately he had used gold or silver springs or bands. To correct any dental irregularity in the front of the mouth, he would pass the band over the labial surface of the teeth, and attach the ends firmly by ligatures to some of the back teeth. This band could be so applied as to force a tooth inward, or draw a tooth outward, by means of ligatures. In short, a tooth could be turned or drawn into any desired position by this appliance, and without material inconvenience to the patient. In a majority of cases, irregularities should be corrected by the canine teeth, for these, as a general rule, are in there proper place in the circle.

Dr. Whitslar asked Dr. Robinson how he would correct an irregularity of the four superior incisors where they closed inside the inferior incisors. Would it not be best to use a plate or blocks to force the teeth into position?

Dr. Robinson replied that by the use of the band or spring the teeth referred to could be brought into proper position much quicker, and with less inconvenience to the patient than by any other appliance.

Dr. C. R. Butler spoke concerning the practice, of some, in extracting teeth for the purpose of making room for correcting dental irregularities. He would like to see an earnest protest throughout the profession against such a practice of sacrificing teeth. To the matter of correcting dental irregularities, he had given some attention. The principal difficulty he had to contend with, was in getting and having control of the patient. The simpliest appliances are generally the best.

After the teeth are once in position a stay-plate or splint should be worn to keep the teeth in place until the healing process is fully completed.

Dr. J. E. Robinson. The first thing to be done in correcting dental irregularities, is to put the teeth in good condition; fill all that need filling.

Dr. Lyder wished to know if it would be safe to move a tooth when the pulp had been destroyed and a large filling had been inserted.

Dr. L. Buffett thought it would not be prudent to meddle with a tooth in that condition.

On motion the association adjourned to meet at nine o'clock a. m.

MORNING SESSION.

Meeting was called to order at nine a. m., by Vice-President J. F. Siddall, and prayer offered by Dr. F. S. Slosson.

Dr. J. E. Robinson, being called upon, presented appliances for correcting dental irregularities. By models, he demonstrated in an interesting manner his *method* of applying bands or springs, a description of which can not well be written out; it must be seen in order to be appreciated.

Dr. F. S, Slosson related a case of correcting the irregularities of the superior lateral incisors where they were inside the centrals, more than the thickness of a tooth. Used but slight pressure, and had no difficulty of bringing them into proper position.

Thought we should deal gently with the teeth; and regarded the simplest applicances as the best. Would never extract the canine teeth for the purpose of making room for others.

A communication was received from Dr. G. R. Thomas, of Detroit, Mich., and read before the association, announcing the death of his youngest daughter, a very interesting child, two years old, as the reason why he could not be present at the meeting of the association. He expressed a deep interest in the matter of replanting and transplanting the natural teeth; and thought great good might result therefrom.

The following was then presented and adopted.

Resolved, That we deeply sympathise with Dr. G. R. Thomas in the affliction which has come upon him and his, in the loss of one of his family; and that we also extend to him our thanks for his kind remembrance and communication to the members of this association.

The next business of the association, was the election of officers for the ensuing year. The result of this election was as follows:

For President, J. F. Siddall, of Oberlin, O.

For Vice-President, J. M. Whitney, of Cleveland, O,

For Rec. Secretary, L. C. Kilsey, of Elyria, O.

For Cor. Secretary, J. Stephan, of Cleveland, O.

For Treasurer, J. E. Robinson, of Cleveland, O.

BOARD OF EXAMINERS.

S. P, Hildreth, of Norwalk, O., L. Buffett, of Cleveland O., and F. S. Whitslar, of Youngstown, O,

On motion a committee consisting of Drs. J. E. Robinson, J. W. Lyder, and J. Stephan was appointed to select delegates to attend the next annual meeting of the American Dental Association which convenes at Philadelphia, August 1st, 1876.

Dr. S. P. Hildreth, chairman of the Board of Examiners, proposed the name of Dr. J. R. Bell as a suitable person to become a member of the association.

On motion Dr. J. R. Bell was elected to membership.

A motion was made and carried that the President appoint three members of the association to prepare essays for the next annual meeting. In compliance with this motion the President appointed the following essayists: Drs. S. P. Hildreth, Chas. Buffett and F. S. Slosson.

The committee appointed to select delegates to the next annual meeting announced as such delegates the following:

Drs. C. R. Butler, J. W. Lyder, David Gibbons, F. S. Whitslar, S. P. Hildreth, R. R. Peebles, C. B. Knowlton and L. C. Kilsey.

On motion, the report of the committee on delegates to the American Dental Association was adopted.

A motion was then made that when this association adjourns, it should adjourn to meet in Cleveland, Ohio, on the first Tuesday in May, 1877. Carried.

Dr. S. P. Hildreth propose the name of J. W. Burrell as a suitable person to become a member of this association.

On motion J. W. Burrell was elected to membership in the association.

The following resolution was then presented and adopted.

Resolved, That the thanks of this association be, and are hereby tendered to Dr. D. R. Jennings, for the use of his rooms during the present sessions of the association.

On motion the association adjourned to meet at two o'clock p. m.

AFTERNOON SESSION.

Meeting called to order at two p. m. by the President elect, J. F. Siddall, who very quietly took his seat evidently intending to proceed at once to business; but the members were in for an inaugural speech, and the President responded to the demand in a very unique manner.

The next subject, The Different Bases for Artificial Teeth was taken up.

Dr. D. F. Wemple remarked, that his experience had been quite extensive in making artificial dentures. Not long ago he concluded to try celluloid as a base for artificial teeth. And to his mind there were two objections to celluloid plates; one was the tendency to warping, the other to discoloring

Dr. Gibbons said he was not pleased with celluloid.

Dr. Newton: Had been quite successful in making celluloid plates. For full sets he was much pleased with celluloid. The discoloring of the plates is some objection. Partial plates, made thin, are liable to warp.

Dr. Knowlton: Used celluloid exclusively for plates. In putting up his cases he used glycerine, and never had any trouble as to the warping of plates. The main objection to a celluloid plate is the offensive smell it has after having been worn for some time.

Dr. Whitslar thought it was high time we express ourselves on this subject of artificial dentures. He was partial to gold plates and when contour was to be restored, was in favor of the continuous gum work. Nothing had ever been used equal to these.

Dr. J. E. Robinson: Had made only one plate on celluloid, and this was giving satisfaction.

Dr. J. M. Whitney said he had been using celluloid for a few years, and was much in favor of it. Celluloid plates could be mended, perfectly. The odor of a celluloid plate after being worn for some time was an objectionable feature.

Dr. D. R. Jenning: Those cases where there is an offensive odor and discoloring of the plates are in the mouths of persons who take no care of their teeth.

There must be some care and skill in putting up celluloid plates as well as in making other artificial dentures.

Dr. Buffett: In regard to warping, he thought celluloid plates not more liable to than rubber. For partial plates, celluloid was hardly strong enough.

Dr. J. W. Burrell said he had used celluloid quite extensively, had never been troubled with the warping of plates; considered partial plates of celluloid as strong as partial plates of rubber. He made the porcelain work a specialty, and regarded that, or plates of gold or silver preferable to any other kind.

President Siddall: Had used celluloid during the present year, and liked it very much; considered it as good as rubber in all respects. He had recently spent some time in Michigan and found that the dentists in that state were using celluloid quite generally and spoke of it as fully equal to rubber.

The President then made the following appointment for executive committee: J. E. Robinson and D. R. Jennings,

On motion Dr. J. M. Whitney was requested to forward his essay to the DENTAL REGISTER for publication. The Secretary was also instructed to furnish a copy of the minutes of the association to the same journal for publication.

After one of the most profitable and interesting sessions the association adjourned to meet in Cleveland, Ohio, on the first Tuesday in May, 1877. L. C. KILSEY, Rec. Sec.

